# Broadly Reactive IgG Responses to Heterologous H5 Prime-Boost Influenza Vaccination Are Shaped by Antigenic Relatedness to Priming Strains

**DOI:** 10.1128/mBio.00449-21

**Published:** 2021-07-06

**Authors:** Jiong Wang, Dongmei Li, Sheldon Perry, Shannon P. Hilchey, Alexander Wiltse, John J. Treanor, Mark Y. Sangster, Martin S. Zand

**Affiliations:** a Department of Medicine, Division of Nephrology, University of Rochester Medical Center, Rochester, New York, USA; b Informatics Core, Clinical, and Translational Science Institute, University of Rochester, Rochester, New York, USA; c Department of Medicine, Division of Infectious Diseases, University of Rochester Medical Center, Rochester, New York, USA; d Department of Immunology, Vaccine Center, University of Rochester Medical Center, Rochester, New York, USA; e Rochester Center for Health Informatics, University of Rochester Medical Center, Rochester, New York, USA; GSK Vaccines

**Keywords:** H5 monovalent influenza vaccine (MIV), hemagglutinin (HA) antigenic distance, influenza virus antibody landscape, original antigenic sin (OAS), HA imprinting

## Abstract

Prime-boost vaccinations of humans with different H5 strains have generated broadly protective antibody levels. However, the effect of an individual’s H5 exposure history on antibody responses to subsequent H5 vaccination is poorly understood. To investigate this, we analyzed the IgG responses to H5 influenza A/Indonesia/5/2005 (Ind05) virus vaccination in three cohorts: (i) a doubly primed group that had received two H5 virus vaccinations, namely, against influenza A/Vietnam/203/2004 (Vie04) virus 5 years prior and A/Hong Kong/156/1997 (HK97) 11 years prior to the Ind05 vaccination; (ii) a singly primed group that had received a vaccination against Vie04 virus 5 years prior to the Ind05 vaccination; and (iii) an H5-naive group that received two doses of the Ind05 vaccine 28 days apart. Hemagglutinin (HA)-reactive IgG levels were estimated by a multiplex assay against an HA panel that included 21 H5 strains and 9 other strains representing the H1, H3, H7, and H9 subtypes. Relative HA antibody landscapes were generated to quantitatively analyze the magnitude and breadth of antibody binding after vaccination. We found that short-interval priming and boosting with the Ind05 vaccine in the naive group generated a low anti-H5 response. Both primed groups generated robust antibody responses reactive to a broad range of H5 strains after receiving a booster injection of Ind05 vaccine; IgG antibody levels persisted longer in subjects who had been doubly primed years ago. Notably, the IgG responses were strongest against the first priming H5 strain, which reflects influenza virus immune imprinting. Finally, the broad anti-H5 IgG response was stronger against strains having a small antigenic distance from the initial priming strain.

## INTRODUCTION

A number of highly pathogenic avian influenza (HPAI) A viruses, such as the H5, H7, and H9 strains, pose a significant threat to cause human pandemics as a result of their fast mutation rate and high pathogenicity (1, 2). To date, there is no evidence of sustained human-to-human transmission of these strains, despite repeated documentation that humans can contract these viruses from infected poultry (3). The first known human H5N1 infection was reported in 1997 during a poultry H5 outbreak in Hong Kong (4). From 2003 to January 2015, a total of 694 laboratory-confirmed human H5 cases were reported across 16 countries, and 58% of those people died as a result (5). Vaccination against future pandemic strains is the most viable path toward mitigating potential outbreaks. However, current H5 nonadjuvanted monovalent influenza vaccine (MIV) formulations are poorly immunogenic (6–10) and generally require a prime and boost strategy in order to achieve protective levels of immunity (11, 12). Interestingly, boosting with nonadjuvanted MIV, even in subjects who had been primed several years prior, led to robust and broad antibody responses to variant H5 MIVs (11). Such prime and boost strategies also appear to be needed for recent RNA vaccines (13) to other non-influenza virus vaccines, and understanding the immunobiology of this phenomenon remains highly relevant.

It has been generally accepted that immunological protection against influenza virus infection is due predominately to antibodies directed against the viral surface hemagglutinin (HA) protein, which is thus the major target of most influenza vaccines (14). A specific language has evolved to describe the potential confounding effects of such exposure on the development of subsequent immunity to influenza. HA imprinting is the initial exposure to an influenza virus strain, first described for childhood H1 influenza, which emerging evidence suggests may protect from subsequent H5 infection (2). However, when a person is sequentially exposed to two related virus strains, they tend to elicit an immune response dominated by antibodies against the first strain to which they were exposed (15, 16). This is true even following a secondary infection or vaccination. This phenomenon has been variously referred to as “original antigenic sin” (OAS), HA seniority, or a negative antigenic interaction (17–19). Thus, the immune response to a new influenza viral infection or vaccination is at least partially shaped by preexisting influenza immunity. Because there is still antigenic overlap between even mostly dissimilar influenza strains, it is critical to understand the antibody responses against antigenically similar virus stains for vaccine development, especially within the context of OAS.

The HA protein is composed of two domains, the highly plastic globular HA1 head domain and the conserved HA2 stalk domain. The hypervariable head domain is believed to be immunodominant, and virus infection or/and vaccination elicits strain-specific neutralizing antibodies primarily targeting this domain, resulting in limited cross-reactivity to divergent virus strains that vary significantly in their HA1 head domain sequences (20). In contrast, antibodies targeting the conserved HA2 stalk domain have been shown to broadly cross-react with multiple influenza viral strains (21). The viruses themselves can be categorized based on the phylogenetic distances of HA sequences. Ten clades of H5 HA (clades 0 to 9) have been identified within the H5N1 virus subtype (22). H5N1 viruses from clades 0, 1, 2, and 7 have the capacity to infect humans (23). These scatter into three distinct antigenic clusters, as determined by antigenic cartography generated by analyzing neutralizing serum antibody levels elicited in mice vaccinated against single influenza virus strains (1). An effective H5 influenza vaccine would ideally induce broad cross-reactivity against all three H5 clades. However, as discussed above, HA imprinting or OAS may impede the generation of broadly cross-reactive H5N1 antibodies if the prime and boost H5N1 vaccine strains reside in different antigenic clusters.

**FIG 1:**
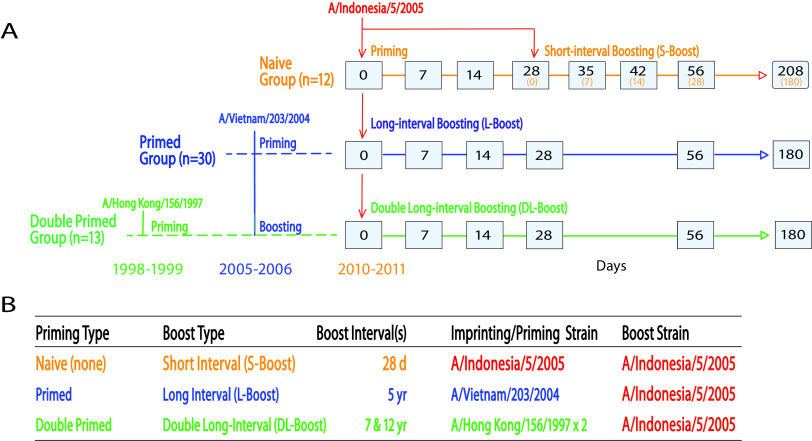
Vaccination strategy. (A) Trial and sampling design. All subjects in the DMID 08-0059 study cohorts were vaccinated with inactivated A/Indonesia/5/2005 (Ind05) intramuscular influenza vaccine. The naive group and short-interval boost (S-boost) group received the Ind05 vaccine on day 0 and short-interval boosting on day 28. The primed long-interval boost (L-boost) group had previously received the inactivated subvirion influenza A/Vietnam/1203/2004 (Vie04) vaccine in 2005 to 2006, and the doubly primed long-interval boost (DL-boost) group additionally received the baculovirus-expressed recombinant influenza A/Hong Kong/156/1997 (HK97) vaccine in 1997 to 1998. Both the L-boost and DL-boost groups also received long-interval vaccination with Ind05 on day 0. Gray boxes indicate serum sampling. (B) Summary of prime and boost strains and groups. d, day.

To address this issue, we reanalyzed serum samples from a previous H5 human vaccine study (DMID 08-0059) (24) using our mPlex-Flu multiplex assay (25) to measure the anti-HA IgG antibodies against all 10 clades (subclades) of H5 influenza virus. During this study (Fig. 1), longitudinal samples were collected prior to and after vaccination with an inactivated influenza A/Indonesia/5/2005 (Ind05) MIV from (i) subjects who had received two prime H5 MIV vaccinations (A/Hong Kong/156/1997 [HK97] in 1997 to 1998 and A/Vietnam/1203/2004 [Vie04] in 2005 to 2006 [the doubly primed long-interval boost {DL-boost} group]), (ii) subjects who had received only one prime Vie04 vaccination in 2005 to 2006 (long-interval boost [L-boost] group), and (iii) subjects in an H5 influenza virus-naive group, who were also given the Ind05 booster 28 days after the prime event (short-interval boost [S-boost] group). The mPlex-Flu assay (25) enables us to simultaneously evaluate the magnitude and breadth of the IgG repertoire directed against HAs from 21 H5 influenza virus strains and 9 other influenza A virus (IAV) strains (H1, H3, H7, H9). We also introduce a novel multiple-dimensional data analysis method named relative antibody landscapes, which enables quantitative analysis of antibody responses to antigenically similar influenza virus strains related to vaccine strains. The relative antibody landscapes method enables analysis of antibody-mediated immunity to a spectrum of HAs after H5 vaccine priming and boosting. This report demonstrates that as the relative antigenic distance between the original priming strain and the new H5 boosting vaccine strain becomes smaller (i.e., the strains are more antigenically similar), the greater the increase in the anti-HA IgG response to the original H5 MIV strain. Thus, in a vaccine response, the original HA imprinting influences vaccine responses occurring significantly later. We discuss the relevance of these findings to the development of influenza vaccines designed to induce broad antibody-mediated protection.

## RESULTS

### Characteristics of subjects.

Prior exposure to the predominant seasonal H1 or H3 influenza strain circulating close to a subject’s birth year can alter H5 or H7 infection and death rates (2, 26). Thus, we first tested for differences in age, as a surrogate for circulating strains, that may alter the antibody levels between the H5 vaccine groups. To assess the birth year-related influenza virus exposure history, we regrouped the study cohorts based on two key birth years, 1968 and 1977, when H3 and H1, respectively, became the dominant circulating influenza A virus strains (Table 1) (2). Subjects without baseline (prevaccination) serum samples were excluded, leaving a total of 55 subjects. The H5-naive subjects (naive group, *n *= 12) and primed subjects (L-boost group, *n *= 30) previously received an inactivated subvirion influenza A/Vietnam/1203/04 (Vie04) virus vaccine in 2005 to 2006 (11). The doubly primed group (DL-boost group, *n *= 13) received the recombinant influenza A/Hong Kong/156/1997 (HK97) virus vaccine in 1997 to 1998 (6) and the Vie04 vaccine in 2005 to 2006. We found no significant difference in birth year distributions between the cohorts (*P* > 0.05; Fisher’s exact test), suggesting that the effects of flu exposure history on the H5 MIV response should be similar across the three groups.

**TABLE 1:** Numbers of subjects stratified by birth year in each cohort of the DMID 08-0059 study^*a*^

Subject group	No. (%) of subjects with the indicated birth yr(s), when the indicated strains were circulating			Total
	<1968, H1 or H2	1968–1977, H3	>1977, H3 and H1	
Naive (short-interval boost [S-boost])	10 (83)	1 (8)	1 (8)	12
Long-interval boost (L-boost)	24 (80)	3 (10)	3 (10)	30
Doubly primed long-interval boost (DL-boost)	11 (85)	2 (15)	0 (0)	13

a Subjects were grouped by birth year based on key years when either H3 or H1 represented the predominant circulating seasonal flu strain, as prior exposure history might influence the antibody responses to the H5 vaccines.

### High anti-H5 IgG responses after long-interval boosting are shaped by the priming vaccine strain.

**FIG 2:**
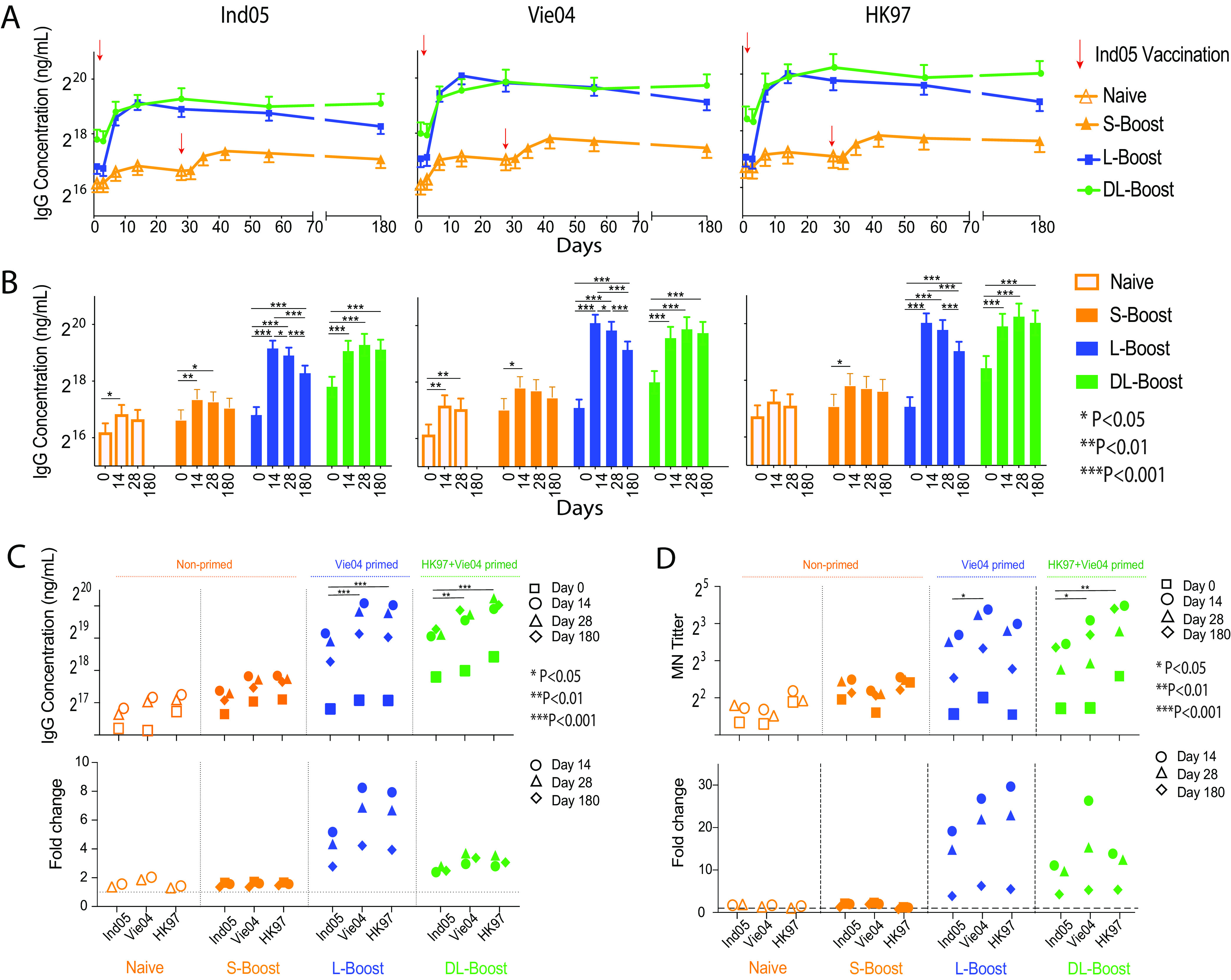
Effects of prior vaccination with the monovalent H5 influenza vaccine (MIV) on multiplex HA antibody responses against three different H5 virus boosting vaccine strains. The mean IgG concentration for each group and standard deviation were estimated by the mPlex-Flu assay. Antibody concentrations were adjusted within the linear mixed-effects models using age at enrollment, gender, ethnicity (Caucasian versus non-Caucasian), dose (two dose levels, 15 and 90 μg), and assay batch (five batches) (27, 28). (A) The dynamic H5 antibody levels against three vaccine strains after MIV H5 vaccination with A/Indonesia/05/2005 (Ind05; clade 2). Four groups are displayed: the prime response (Naive; orange, unfilled symbols) and short-interval boost response (S-boost; orange filled symbols) of naive subjects; the long-interval boost response (L-boost; blue filled symbols) after one dose of Ind05 MIV in subjects primed by Vie04 MIV 5 years previously; and finally subjects who were double-primed with Vie04 (5 years previously) and A/Hong Kong/156/1997 (HK97; clade 0) HK97 (12–13 years previously) responses (DL-boost; green filled symbols). (B) Comparison of antibody responses between time points in the same groups for each vaccine strain. (C) Antibody concentrations against each vaccination strain and fold changes from levels on day 0, grouped by study cohort. (D) Antibody titers for microneutralization (MN) against each vaccination strain and fold changes from levels on day 0, grouped by study cohort. The original MN assay data were reanalyzed with linear mixed-effects modeling, as described above. Shown are the geometric means of titers. *, *P* < 0.05; **, *P* < 0.01; ***, *P* < 0.001. Linear contrasts within the linear mixed-effects model framework were used to conduct the statistical comparisons.

Using a 48-HA mPlex-Flu assay panel, we observed that IgG levels against the HA of influenza A/Indonesia/5/2005 (Ind05), Vie04, and HK97 were very low in the naive group and about 2-fold higher in the short-interval boosting (S-boost) group, whose members were boosted after 28 days (Fig. 2A and B). In both primed groups (L-boost and DL-boost), however, inactivated Ind05 MIV induced ∼5-fold-higher vaccine-specific antibody levels by 14 days postvaccination. Anti-Vie04 and -HK97 IgG levels increased ∼7- to 8-fold, also peaking at 14 days in both primed groups (Fig. 2). While both primed groups had higher preexisting (day 0) anti-H5 IgG levels, their IgG response kinetic curves against the vaccine strains were similar. These differences result in a relative increase in the DL-boost group’s anti-HA antibody levels peaking at 3.5-fold (Fig. 2D), even though the postboost IgG levels were similar in the S- and DL-boost groups. In both groups, anti-H5 HA antibodies levels remained high for over 6 months. These results are consistent with the previous findings that nonadjuvanted MIVs are poorly immunogenic in naive subjects (6–10) and that long-interval boosting with H5 antigenic variant MIVs elicits significant and robust antibody responses (11, 24). However, this is the first report to show differences in antibody response induced by single versus double long-interval MIV boosting.

Importantly, we also found that the Ind05 MIV elicited robust antibody responses against the two previous priming H5 strains (Vie04, HK97) in both vaccine groups and that the anti-HA IgG responses shared similar kinetic patterns. Interestingly, the Ind05 MIV elicited higher levels of IgG antibodies to Vie04 and HK97 than to Ind05. In order to directly compare the effects of the priming virus strain, we plotted the concentrations of anti-H5 HA by groups, shown in Fig. 2C, and the fold change of antibody concentrations against three vaccine strains of the different groups (Fig. 2D). The results revealed high antibody levels against the HA of Vie04 in the L-boost group and that of HK97 in the DL-boost group, which were the first H5 viral strains against which subjects were vaccinated. These results might be interpreted as indicative of HA imprinting (15, 16), in which subjects generate a robust antibody response against the H5 influenza virus strain to which they were first exposed by infection or vaccination, and subjects maintain this response over their entire lifetime (26).

To confirm the protective activities of the higher level of long-lasting antibodies in the L-boost and DL-boost groups, we reanalyzed the hemagglutination inhibition (HAI) and microneutralization (MN) data from the DMID 08-0059 study using generalized linear mixed-effects models with identity link functions, as we previously described (27, 28). The results confirmed that all three H5 MIV strain vaccines induced serum with a virus-neutralizing capacity that could protect cells from viral infection (Fig. 2D and see Fig. S8 in the supplemental material).

10.1128/mBio.00449-21.9 FIG S8 Prior vaccination with a monovalent influenza vaccine (MIV) increased the serum titers of hemagglutination inhibition (HAI) and microneutralization (MN) antibody responses against three antigenically drifted virus vaccine strains, including the new vaccine strain Ind05 (clade 2) and the previous MIV strains Vie04 (clade 1) and HK97 (clade 0). Naive subjects (Unprimed) received the MIV Ind05 strain and were subsequently boosted at day 28 with the same strain. A previously primed group had received the MIV Vie04 5 years prior (Primed) and then received a single dose of Ind05. The previously doubly primed group received MIV Vie04 and HK97 (Multiple). The mean IgG concentration for each group and standard deviation were estimated by linear mixed-effects models, with group, day, and group-day interactions used to fit the data for each H5 vaccine strain. Covariates adjusted in the linear mixed-effects models included the following: age at enrollment, gender, ethnicity (Caucasian versus non-Caucasian), dose (two dose levels, 15 and 90 μg), and batch (five batches). *, *P* < 0.05; **, *P* < 0.01; ***, *P* < 0.001. Linear contrasts within the linear mixed-effects model framework were used to do the statistical testing. Download FIG S8, EPS file, 1.3 MB. Copyright © 2021 Wang et al. 2021 Wang et al. https://creativecommons.org/licenses/by/4.0/ This content is distributed under the terms of the Creative Commons Attribution 4.0 International license.

### Relative antigenic response landscapes of H5 MIV HAs.

Our results also raised another fundamental question: does the magnitude of the imprinted recall response to the original priming H5 HA correlate with the antigenic distance between the HAs of the prime and boost strains? We hypothesized that the antigenic distance between the vaccine strain and a target H5 HA is inversely correlated with the cross-relativity of the antibody response induced by the H5 MIV. In other words, smaller antigenic distances from the first influenza virus strain (imprinting strain) produce larger IgG responses. To answer this question, we performed antigenic cartography to quantitatively evaluate the antigenic distances between H5 clades and subclades.

Recombinant H5 HA proteins were expressed and purified. Strains were chosen to cover all 10 H5 clades (0 to 9) and subclades and 4 new H5 avian strains (Cl4.4.4.3) isolated in the United States (Table S1 and Fig. S1). Antibody reactivity to these strains was plotted against mouse anti-H5 HA IgG serum reactivity generated utilizing a monovalent DNA vaccination approach (Fig. S2A). We thus generated a comprehensive antigenic distance matrix between 17 H5 influenza virus strains and each of 21 H5 and 9 other influenza virus strains using the mPlex-Flu assay. The individual antibody levels against H5 viruses are shown as mean fluorescence intensity (MFI) units at specific dilutions, with the dilution factors being normalized using a generalized linear model with an identity link function for the serum samples. We used classical multidimensional scaling (MDS) (29) to project relative distances between strains into 2 dimensions, and the matrix data were created by calculating a Euclidean distance matrix from two-dimensional coordinates. Finally, we used a modification of the approach of Smith et al. (30) to visualize the antigenic distance between influenza virus HAs (1, 30) (Fig. S2C). This approach accounts for the continuous nature of the mPlex-Flu assay data and the consistent range of estimated strain-specific binding (27, 28), yielding the same results as antigenic cartography. The antigenic distance matrix was also generated from the above multiplex data of the mPlex-Flu assay using the single-virus DNA vaccine antisera (Fig. S3).

10.1128/mBio.00449-21.2 FIG S1 HA protein characters of 35 influenza virus A strains in the mPlex-Flu assay. (A) The phylogenetic tree was generated using HA amino acid sequences of the 35 influenza A virus strains obtained from the phylogenic tree maker on the Influenza Research Database Website (https://www.fludb.org/brc/home.spg?decorator=influenza). (B)SDS-PAGE gel image of purified HA proteins of H5 influenza viral strains. (C) High-performance liquid chromatography (HPLC) analysis results of four representative HA proteins flowing through the Biosep-SEC-s4000 columns with the Bio-Rad protein standards. Download FIG S1, PDF file, 2.9 MB. Copyright © 2021 Wang et al. 2021 Wang et al. https://creativecommons.org/licenses/by/4.0/ This content is distributed under the terms of the Creative Commons Attribution 4.0 International license.

10.1128/mBio.00449-21.3 FIG S2 Antigenic cartography is generated with a mouse DNA vaccination model. (A) Mouse DNA vaccination strategy. (B) Heat map of the multiple-dimensional antibody data generated by the mPlex-Flu assay. Each mouse polyclonal antiserum was induced by DNA vaccination with a DNA plasmid encoding HA proteins, and the antibody levels in the sera were estimated by the mPlex-Flu assay. (C) Antigenic cartography of 36 influenza A virus strains assessed by the mPlex-Flu assay with the multiple-dimensional scaling (MDS) method. Download FIG S2, EPS file, 2.9 MB. Copyright © 2021 Wang et al. 2021 Wang et al. https://creativecommons.org/licenses/by/4.0/ This content is distributed under the terms of the Creative Commons Attribution 4.0 International license.

10.1128/mBio.00449-21.4 FIG S3 Heat map matrix of the antigenic distances between the 21 H5 influenza virus strains. The three vaccination strains are highlighted with red arrows. Download FIG S3, EPS file, 2.1 MB. Copyright © 2021 Wang et al. 2021 Wang et al. https://creativecommons.org/licenses/by/4.0/ This content is distributed under the terms of the Creative Commons Attribution 4.0 International license.

10.1128/mBio.00449-21.10 TABLE S1 mPlex-Flu assay panel of seasonal influenza viruses, H5 clades, and subclades. Download Table S1, PDF file, 0.1 MB. Copyright © 2021 Wang et al. 2021 Wang et al. https://creativecommons.org/licenses/by/4.0/ This content is distributed under the terms of the Creative Commons Attribution 4.0 International license.

**FIG 3:**
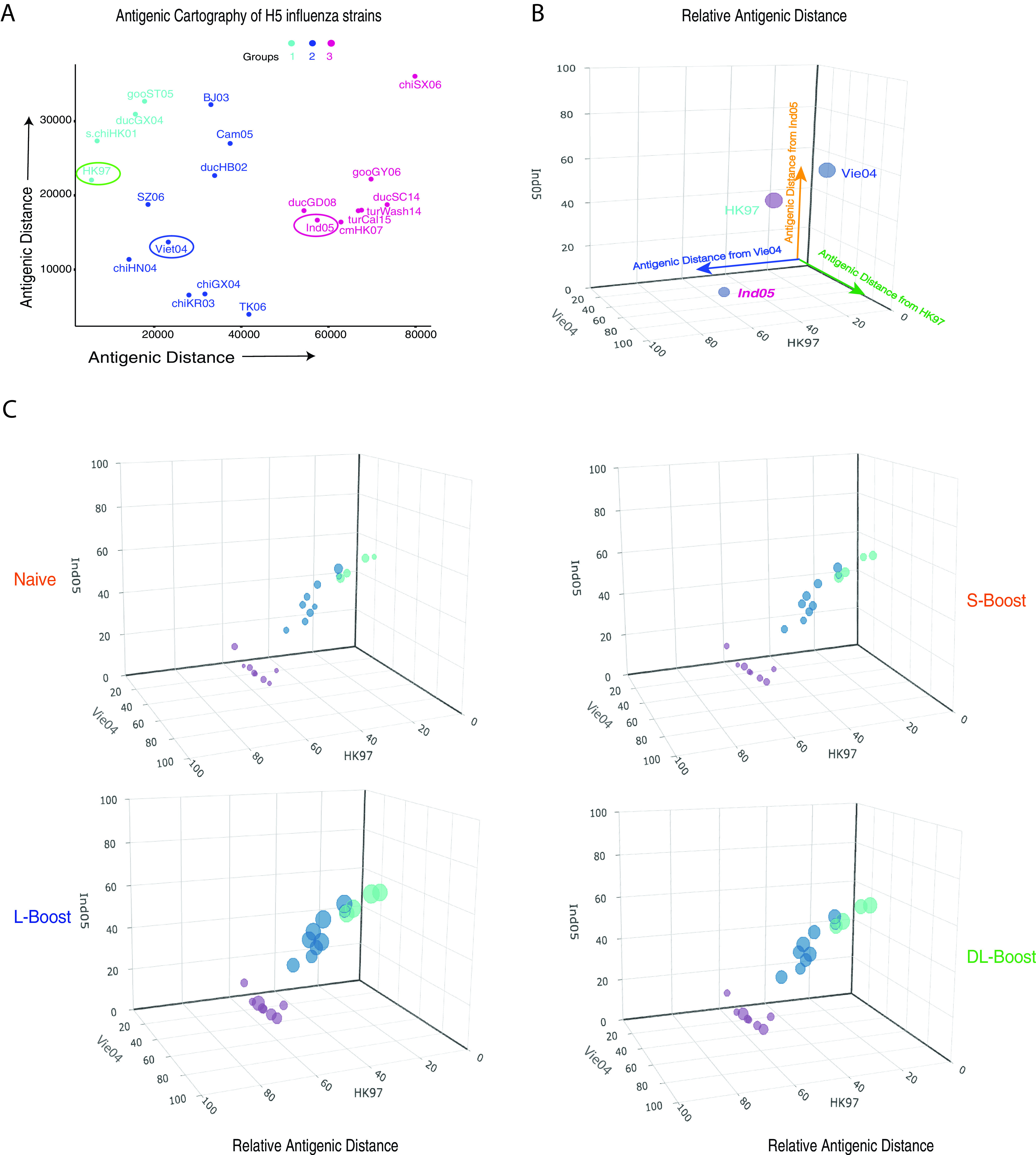
HA antibody responses were plotted against the related antigenic distances in each MIV strain of different study cohorts. (A) Antigenic cartography of 21 H5 influenza virus strains generated by mPlex-Flu assay of antisera against 17 anti-H5 influenza viruses and plotted using classical multidimensional scaling (MDS; see Materials and Methods). The three vaccine strains are circled. (B) We then used three-dimensional (3D) plots to show the relative antigenic distances of all mPlex-Flu target HAs to the three vaccine strains HK97 (clade 0), Vie04 (clade 1), and Ind05 (clade 2). (C) IgG responses of subjects in the DMID 08-0059 study to 21 H5 strains plotted in 3D bubble plots. The relative antigenic distances of the 21 H5 strains assayed were plotted against their antigenic distance to each of the three MIV strains to determine their 3D antigenic cartography. The bubble sizes represent the concentration (10^4^ ng/ml) of IgG against an H5 influenza virus at day 14 after MIV boosting. (A) Using unsupervised hierarchical clustering, three H5 antigenic groups were identified. Interactive 3D bubble plots can be accessed through the following links: for the prime group, http://rpubs.com/DongmeiLi/565996; S-boost group, http://rpubs.com/DongmeiLi/565998; L-boost group, http://rpubs.com/DongmeiLi/565989; and DL-boost group, http://rpubs.com/DongmeiLi/565994.

In order to show the relative antigenic distances between individual HAs and the H5 MIV strains (Fig. 3B), we plotted the distance of each H5 HA from those in the 3 vaccine strains: HK97 (*x* axis), Vie04 (*y* axis), and Ind05 (*z* axis). Each marker diameter represents the magnitude of the IgG concentration 14 days after MIV boosting. This allowed visualization of the magnitude of the antibody response against specific H5 HAs associated with the antigenic distances with respect to both the prime and boost vaccine strains in the different cohort groups. The same diagram allowed visualization of H5 vaccine strain relative distances from other H5 strains. Naive subjects had low anti-HA IgG levels against all H5 strains after priming and short-interval boosting with MIV. However, the L-boost and DL-boost groups had significantly enhanced antibody responses after 14 days, with higher IgG responses to H5 strains in the Vie04 and HK97 cluster groups than to those in the Ind05 cluster MIV group, which are antigenically similar to the strain of the more recent MIV (Fig. 3C). These data clearly show the relationship between the anti-HA IgG antibody response and the antigenic distances from HAs in the reference strains: higher cross-reactive antibody levels are elicited against the HAs from strains in the same cluster group as the first priming virus strain.

### Long-term boosting of MIV elicits heterogeneous IgG responses against all H5 clade/subclades, which are correlated with the antigenic distances to the first primed virus strains.

**FIG 4:**
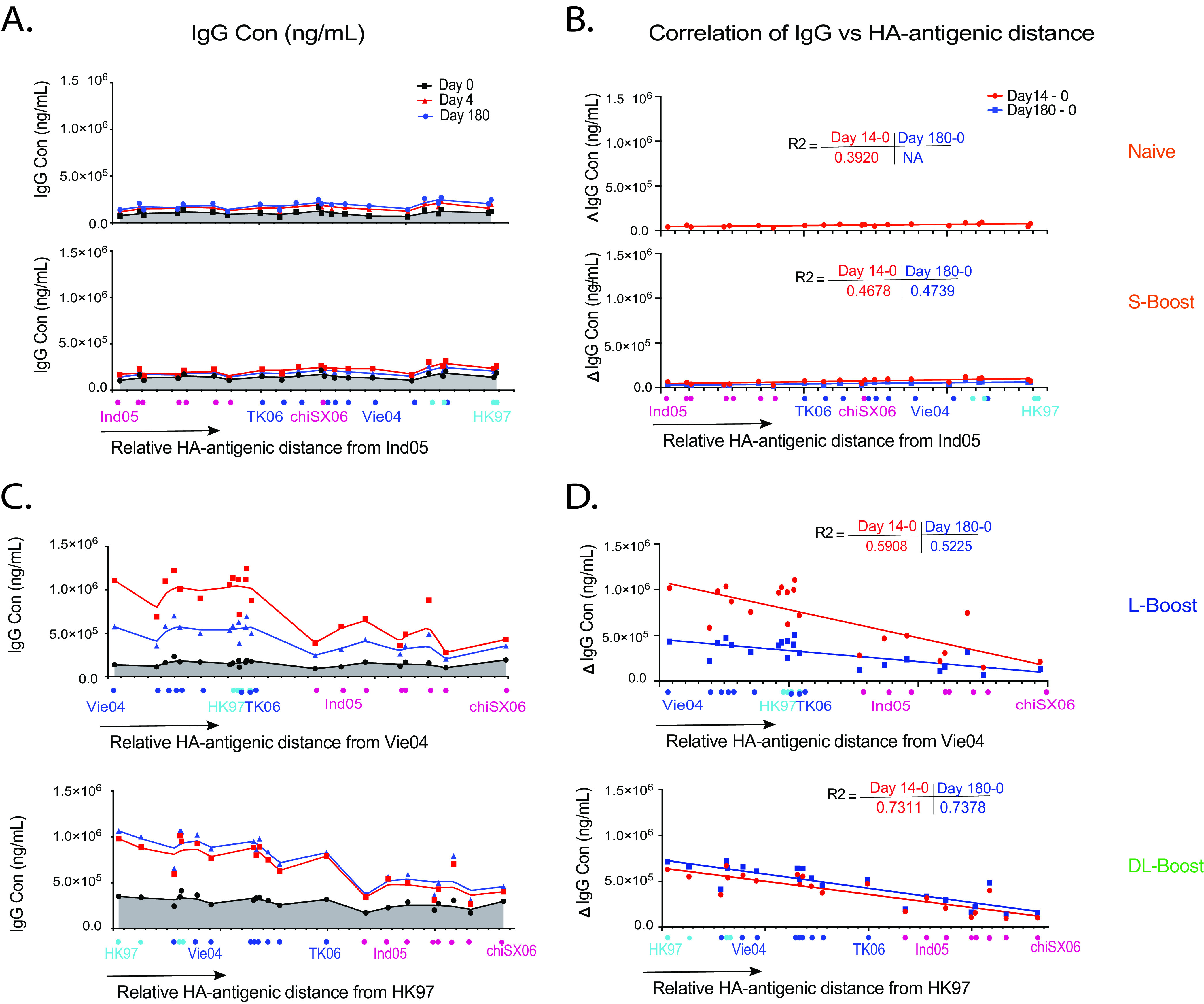
Relative HA antibody landscapes, anti-HA IgG levels, and relative antigenic distances from vaccine strains. (A) Relative HA antibody landscapes of H5 virus strains as a function of the relative HA antigenic similarity distance from the vaccination strain Ind05 for the naive group and S-boost group (see Materials and Methods). (B) Correlation of the HA antibody responses of the naive and S-boost groups to the HA antigenic distance from the vaccine strain HAs. The coordinates of each H5 strain result represent the relative antigenic distance of H5 HA*_i_* (an individual H5 strain in the panel) to the vaccine strain HA on each axis. (C) Relative HA antibody landscapes for each group using the relative HA antigenic distance from the H5 reference strain Vie04 (clade 1) or HK97 (clade 0). (D) Correlation between the HA antibody response and the HA antigenic distance to the imprinting (first-exposure) H5 strain Vie04 for the L-boost group or HK97 for the DL-boost group. The change in IgG concentration (ΔIgG Con) is the difference between the pre-and postvaccination anti-HA antibody IgG concentrations.

We next generated antigenic landscape plots (26) to visualize the magnitude of serological responses in relation to the antigenic distance between the vaccine strain HA and the H5 HAs in the mPlex-Flu panel. We first focused on the relationship between the magnitude of a boosted IgG response and the antigenic distance between the boost HA and the HAs in the three H5 vaccine strains. To this end, IgG antibody concentrations against 21 H5 strains were measured by the mPlex-Flu assay for each cohort on days 9, 14, and 28, which were plotted against their relative antigenic distances from HA in Ind05 (Fig. 4A and B), Viet04 (Fig. 4C and D), and HK97 (Fig. 4E and F). Correlation test results are given in the figure insets, and all data are presented in Fig. S4, S5, and S6.

10.1128/mBio.00449-21.5 FIG S4 Correlations between the HA antibody responses and the HA antigenic similarity of A/Hong Kong/156/1997 (HK97) to 21 H5 influenza virus strains. (A) The HA antibody response landscape-like plots of each group using the relative HA antigenic distance of HK97 (clade 0) as the reference strain (see Materials and Methods). The *x* axis is relative antigenic distance, the *y* axis is the IgG antibody response, and the spots were linked by LOWESS fit spline curve (Prism 8 software). (B) Correlation of the HA antibody response to the HA antigenic distance. The change in antibody concentration pre- and postvaccination versus the relative HA antigenic distance of Vie04. The *R*^2^ values were calculated with simple linear regression analysis (Prism 8 software). Download FIG S4, EPS file, 1 MB. Copyright © 2021 Wang et al. 2021 Wang et al. https://creativecommons.org/licenses/by/4.0/ This content is distributed under the terms of the Creative Commons Attribution 4.0 International license.

10.1128/mBio.00449-21.6 FIG S5 Correlation between the HA antibody responses and the HA antigenic similarity between A/Vietnam/1203/2004 (Vie04) and 21 H5 influenza virus strains. (A) HA antibody response landscape-like plots of each group using the relative HA antigenic distance of Vie04 (clade 1) as the reference strain (see Materials and Methods). The *x* axis is the relative antigenic distance, the *y* axis is the IgG antibody response, and the spots were linked by LOWESS fit spline curve (Prism 8 software). (B) Correlation of the HA antibody responses to the HA antigenic distance. The change in antibody concentration pre- and postvaccination versus the relative HA antigenic distance of Vie04. The *R*^2^ values were calculated with simple linear regression analysis (Prism 8 software). Download FIG S5, EPS file, 1 MB. Copyright © 2021 Wang et al. 2021 Wang et al. https://creativecommons.org/licenses/by/4.0/ This content is distributed under the terms of the Creative Commons Attribution 4.0 International license.

10.1128/mBio.00449-21.7 FIG S6 Correlation between the HA antibody responses and the HA antigenic similarity of A/Indonesia/5/2005 (Ind05) to 21 H5 influenza virus strains. (A) HA antibody response landscape-like plots of each group using the relative HA antigenic distance of Ind05 (clade 1) as the reference strain (see Materials and Methods). The *x* axis is the relative antigenic distance, the *y* axis is the IgG antibody response, and the spots were linked by LOWESS fit spline curve (Prism 8 software). (B) Correlation of the HA antibody responses to the HA antigenic distance. The change in antibody concentration pre- and postvaccination verse the relative HA antigenic distance of Vie04. The *R*^2^ values were calculated with simple linear regression analysis (Prism 8 software). Download FIG S6, EPS file, 1 MB. Copyright © 2021 Wang et al. 2021 Wang et al. https://creativecommons.org/licenses/by/4.0/ This content is distributed under the terms of the Creative Commons Attribution 4.0 International license.

We found that the immune responses in the naive and S-boost groups were very weak, and since subjects in these groups were exposed only to the Ind05 MIV strain, we made antigenic landscapes (26) using Ind05 as the reference influenza virus strain. The relative antigenic landscapes for these two groups at days 0, 14, and 180 are shown in Fig. 4A and B. Similarly, the serological responses of the L-boost and DL-boost groups after boosting were plotted against the antigenic distance from HAs in Vie04 and HK97, shown in Fig. 4C and D. Note that the antigenic distance between the cognate vaccine strain and itself is zero (e.g., Vie04 – Vie04 = 0). The Ind05 MIV showed very low antigenicity in both naive subject groups. Changes in IgG concentration (Δ*IgG* = [*IgG_t_*] – [*IgG*_day 0_], where *IgG_t_* is the IgG concentration at time point *t*) were not correlated with antigenic distance (*P* = 0.014 and 0.020). However, Ind05 MIV boosting showed higher antibody responses to HAs from strains with a smaller antigenic distance in both the L-boost (*R*^2^ = 0.57) and DL-boost (*R*^2^ = 0.73) groups. These results support our hypothesis that that the imprinting of primed individuals is highly correlated with the antigenic distance related to the priming strains for long-interval H5 vaccination (Fig. 4).

### Long-interval boosting with H5 MIV induces broadly heterosubtypic antibody responses against group 1 influenza viruses.

**FIG 5:**
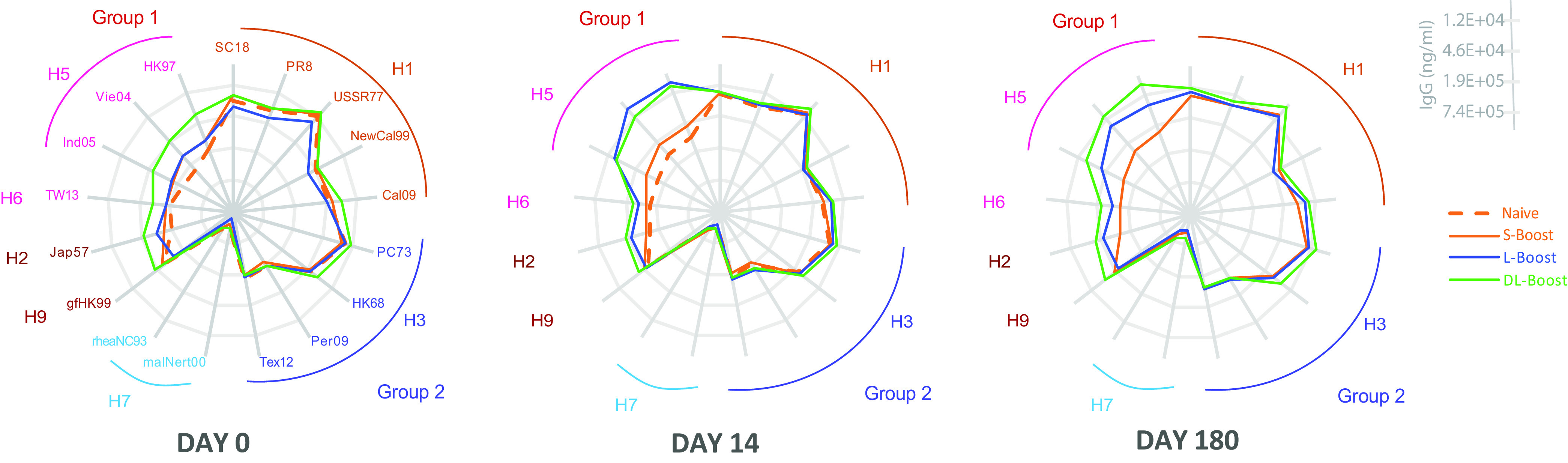
Human heterosubtypic IgG antibody responses elicited by H5 MIV. The IgG antibody responses induced by the H5 influenza vaccine against previously circulating or vaccine virus strains (H1, H2, H3, H6, H7, H9) were measured by the mPlex-Flu assay prevaccination (day 0) and postvaccination (days 14 and 180).

To assess the breadth of heterosubtypic immunity generated by the H5 MIV prime and boost strategy, including IgG reactive against other influenza virus HAs, we estimated antibody cross-reactivity to select group 1 (H1, H2, H5, H6, and H9) and group 2 (H3, H4, H7) HAs (Table S1) using the mPlex-Flu assay (Fig. 5). In all subjects, we detected high preexisting anti-H1 HA subtype IgG levels against older (A/South Carolina/1/2018 [SC18], A/Puerto Rico/8/1934 [PR8]) and newer (A/New Caledonia/20/1999 [NewCall99], A/California/07/2009 [Cali09]) strains. However, these anti-HA levels were not significantly affected by H5 MIV vaccination (Fig. S7A). In addition, we found dramatic increases in anti-HA IgG levels targeting other group 1 influenza viruses (e.g., H2, H6) that had lower baseline levels than those against influenza group 2 (H1, H3) subtype virus HAs.

10.1128/mBio.00449-21.8 FIG S7 The IgG concentrations of group 1 and 2 influenza virus strains were estimated by the mPlex-Flu assay in the DMID 08-0059 study. The mPlex-Flu assay estimated the mean IgG concentration for each group and the standard deviation. Then the antibody concentrations were adjusted within the linear mixed-effects models, which included the following: age at enrollment, gender, ethnicity (Caucasian versus non-Caucasian), dose (two dose levels, 15 and 90 μg), and batch (five batches). (A) The mPlex-Flu assay estimated the antibody concentrations of group 1 influenza virus strains (including five human H1 strains, one of which was an H2, H6, or H9 strain). (B) The antibody concentrations against group 2 influenza A virus strains (including four H3 and two H7 strains) were estimated by the mPlex-Flu assay. Download FIG S7, EPS file, 1.6 MB. Copyright © 2021 Wang et al. 2021 Wang et al. https://creativecommons.org/licenses/by/4.0/ This content is distributed under the terms of the Creative Commons Attribution 4.0 International license.

Further analysis demonstrated that post-H5 vaccination IgG reactivity across influenza virus strains was inversely correlated with both phylogenetic and antigenic distances between the strains, especially the stalk regions. Based on phylogenetic distance, the gene sequence of H6 is closer to that of H5 than that of H9 (20). Similarly, the gene sequence of H2 is closer to that of H5 than to those of H6 and H1 (Fig. S1A). In addition, we found that IgG responses induced by H5 MIV against HA of A/Japan/305/1957 (Jap57, H2) were significantly higher than those against A/Taiwan/2/2013 (TW13, H6) and A/guinea fowl/Hong Kong/WF10/1999 (gfHK99, H9) (Fig. 5; Fig. S7A). The last two strains have stalk regions phylogenetically and antigenically distant from the H5 clade stalk. We also found that, in both primed groups, H5 MIV elicited cross-reactive anti-H2 IgG responses in naive subjects, with a higher peak and a more sustained duration than in the naive subjects. Those responses were stronger than those against H6 and H9 HAs. No significant changes were detected in IgG levels against H3 and other group 2 influenza viruses (Fig. S7B). Together, these findings also support the hypothesis that cross-strain, anti-HA antibody responses are highly correlated with phylogenetic similarity to and inversely correlated with antigenic distance from the vaccine strain.

### Long-interval boosting elicits IgG antibodies against the HA head domain.

The HA stalk domain is highly conserved within influenza virus phylogenetic groups, and stalk-reactive antibodies have been hypothesized to be the major contributors mediating the cross-reactivity of anti-HA IgG antibodies across group 1 (31) strains. However, broadly cross-reactive neutralizing antibodies against the HA head domain have recently been identified and may also contribute to this phenomenon (reviewed in reference 32). Thus, we next measured the change in the relative proportions of head- versus stalk-reactive IgG within the H5 boosting group.

**FIG 6:**
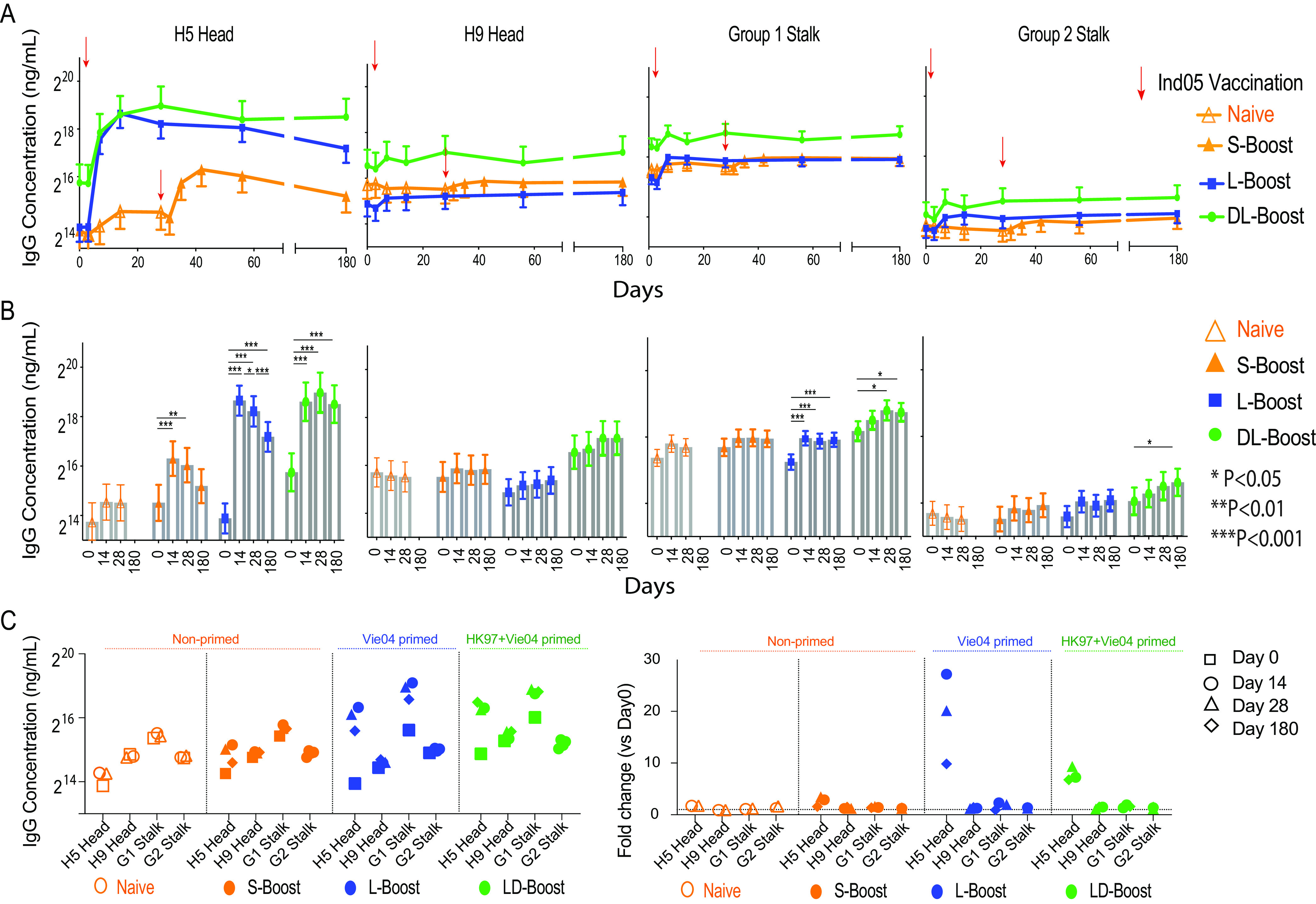
Head- and stalk-reactive IgG responses induced by the human MIV H5 vaccine. (A) Kinetic profile of the IgG responses against the HA head or stalk domain estimated by the mPlex-Flu assay. (B) Comparison of concentrations of each H5 HA-specific antibody prevaccination (day 0) and postvaccination (14, 28, and 180 days). Linear contrasts within the linear mixed-effects model framework were used for statistical testing (*, *P* < 0.05; **, *P* < 0.01; ***, *P* < 0.001). (C) Comparison of anti-HA IgG concentrations between HAs, including antibodies against chimeric cH9/1 HA (group 1 stalk-reactive antibodies; G1 Stalk) and cH4/7 HA (group 2 stalk-reactive antibodies; G2 Stalk).

H5 head (HA1)-specific IgG levels were measured using beads coupled with the Ind05 head domain only. Anti-stalk IgG was measured using chimeric cH9/1 and cH4/7 proteins to estimate, respectively, group 1 and group 2 stalk-reactive antibodies (33–35). The results demonstrate that short-interval boosting can induce an ∼2-fold increase in anti-H5 head IgG levels in naive subjects (Fig. 6). In addition, significant increases in head-specific IgG were also detected in the L-boost group: 27-fold (14 days), 20-fold (28 days), and 10-fold (180 days). Examining the DL-boost group, ∼7- to 8-fold increases were observed at 14, 28, and 180 days after vaccination. High levels of group 1 stalk-reactive IgG were found in both boosting groups. However, these increases accounted for a <2-fold overall change in IgG levels, primarily because these stalk-reactive IgG antibodies were present at relatively high levels prior to vaccination. We did not observe any significant postvaccination increases in group 2 stalk-reactive antibody levels regardless of test groups. Overall, our results suggest that broadly cross-reactive IgG against H5 influenza virus HAs or the phylogenetic group 1 HAs are most likely mediated by conserved epitopes on the head domain of HA as opposed to the stalk domain.

## DISCUSSION

Two major impediments to universal flu vaccine development are the constant antigenic changes of influenza viruses and that the human antibody response is shaped by prior influenza virus exposure history (36). In addition, vaccination strategies for emergent influenza viruses need to take into account both the vaccination schedule and the ability of HA imprinting to hinder immune responses to new antigens. Antibody-mediated immune responses to new HA antigens are generally weak after the priming vaccination and require further boosting to elicit adequate titers for infection prevention. This phenomenon can be leveraged if the subject has been primed by exposure to HA antigens, by prior infection, or by vaccination against H1 or H3 influenza viruses that are antigenically distant from emergent strain HAs (heterosubtypic immunity).

The antigenic distance between two virus strain HAs can be calculated empirically or experimentally. Empirically, antigenic distance is correlated with the difference between surface protein sequences of HA (e.g., edit distance, Damerau-Levenshtein distance). Experimentally, it can be derived by calculating the *n*-dimensional distance between the immune reactivity of sera from a subject vaccinated with a single virus against a panel of other HAs from disparate virus strains (36). As we have previously shown (34), the smaller the antigenic distance between the prime and boost HAs, the stronger the postboost vaccination increase in vaccine-specific anti-HA IgG levels.

In this study, we also analyzed changes in multidimensional anti-H5 HA IgG responses after vaccination and boosting using a modification of the antibody landscape method (26), a variant of antigenic cartography (30). We initially analyzed anti-HA IgG antibody levels against a comprehensive panel of H5 clade/subclade HAs as a function of the relative antigenic distance to the reference vaccine HA. We call this multidimensional measure the relative antibody landscape (Fig. 4A and C). This novel method, combined with multiplex serum IgG measurements, allows an analysis of the breadth of the antibody response as a function of the antigenic distance from the vaccine strain. Our results using the relative antibody landscape method show that the anti-H5 HA IgG responses elicited by boosting in both primed groups are highly correlated with the antigenic distance between the priming and boosting H5 vaccine strains. These findings provide further evidence for HA antigenic imprinting in H5 influenza vaccination. Most significantly, we demonstrate that relative antibody landscape methods can be used to analyze the effects of previous HA antigen exposure on vaccine responses, allowing for quantitative analysis of antigenic imprinting.

Our work also demonstrates that long-interval boosting augments H5 vaccine-induced immunity. Studies using variants of the H5 MIVs have shown that long-interval prime-boost strategies, on the order of 4 to 8 years between vaccinations, result in robust and durable antibody responses (11) to what are relatively poorly immunogenic vaccine components (6, 7, 24). Intermediate intervals of 6 to 12 months between priming and boosting with H5 variants significantly increases antibody responses (37, 38), compared to 8 weeks or less. One potential mechanism for these results is a time-dependent increase in long-lived memory B cells, which may take 2 to 4 months after vaccine priming (39) before these memory B cells can respond rapidly to long-interval boosting (40). Studies showed that an adjuvanted H5 MIV used in short-interval boosting also significantly increased the immunogenicity of vaccines (41–44) and indicated that prime-boost vaccination induced the monoclonal antibodies that largely recognized the HA head region of the H5 MIV strain (45). Significant additional work is necessary to define the optimum prime-boost interval for robust responses.

Our results also support the hypothesis that long-interval boosting increases antibody responses targeting the HA head domain, rather than the stalk. Recently, several broadly neutralizing antibodies (bNAbs) have been identified from both infected and vaccinated human subjects that target the hypervariable HA head domain, including C05 (46), 5J8 (47), CH65 (48), and CH67. These bNAbs exhibit considerable neutralizing breadth within the H1 (46–48) and H3 (49) influenza virus subtypes. Such bNAbs are thought to bind highly conserved regions on the sialic acid receptor binding site (RBS) in the HA head domain, explaining their ability to broadly neutralize viral binding from different subtypes (48, 50). As the head domain is known to be immunodominant in the induction of strong antibody responses, broadly head-reactive antibodies may be the major mediator of cross-reactive immunity across subtypes or heterosubtypes. Our results are also consistent with recent work that found rapid activation and expansion of preexisting memory B cell responses to the conserved epitopes on the HA stalk and head domains after long-interval prime-boost vaccination with H7N9 (39).

Finally, our results contribute further to a framework for thinking about influenza vaccine development strategies. The aspirational goal of an influenza vaccine is to create long-lasting protective immunity to a wide spectrum of influenza viruses. In such cases, future exposure, via infection or vaccination, may occur years after the initial priming and imprinting event. Our work demonstrates that the long-interval prime-boost strategy for H5 vaccination induces long-lasting cross-reactive antibodies against conserved regions on the HA1 head domain. This may help in universal influenza vaccine development, not for a single vaccine but as a long-interval boost strategy to generate cross-reactive antibodies to recognize the conserved sites on HA1 head domain.

In conclusion, we used a multiplex antibody assay and a novel antibody landscape method to analyze antibody-mediated immunity to various HAs after H5 vaccine priming and boosting. These methods quantitatively account for the antigenic distances between the vaccine and other strain HAs. This new approach demonstrated that anti-H5 IgG antibody responses elicited by boosting are highly correlated with the antigenic similarity between the priming and boosting H5 vaccine strains, providing evidence for OAS and HA imprinting within the context of H5 vaccination.

## MATERIALS AND METHODS

### Human subject ethics statement.

This subanalysis study was approved by the Research Subjects Review Board at the University of Rochester Medical Center (RSRB approval number RSRB00012232). Samples were analyzed under secondary-use consent obtained previously as part of a prior clinical trial (24). All research data were coded by sample IDs in compliance with the Department of Health and Human Services’ regulations for the protection of human subjects (51).

### Samples and data.

Serum samples for the multiplex assay were obtained from a prior clinical trial, DMID 08-0059 (Fig. 1) (24). Subjects without prevaccination serum samples (day 0 baseline) were excluded. All subjects in the three cohorts were inoculated with inactivated A/Indonesia/5/2005 (Ind05) vaccine. H5-naive subjects (*n *= 12), who were healthy adults not at risk for H5 exposure and with no H5 vaccination history, received 2 identical Ind05 vaccinations separated by 28 days. Primed subjects (*n *= 30) previously received the inactivated subvirion A/Vietnam/1203/2004 (Vie04) vaccine in 2005 to 2006 (11). The doubly primed group (*n *= 13) had received both the recombinant A/Hong Kong/156/1997 (HK97) vaccine in 1997 to 1998 (6) and the Vie04 vaccine in 2005 to 2006. Serum samples were collected before vaccination (day 0) and on days 7, 14, 28, 56, and 180 after vaccination. Serum samples were collected from the naive group subjects on days 7, 14, and 28 days after the second immunization. All data from the mPlex-Flu, HAI, and MN assays were adjusted for dose difference using linear mixed-effects models, as previously described (27, 28).

### mPlex-Flu analysis.

We estimated the concentrations of anti-HA IgG antibodies against a panel of 45 HA antigens from influenza viruses using the mPlex-Flu assay, as described previously (25, 33). All influenza HA sequence identifiers used are listed in Table S1 in the supplemental material, and the HA genetic distance (phylogenic tree) is shown in Fig. S1A. The panel recombinant HA proteins were expressed by the baculovirus system and purified by Ni^+^ affinity column selection as previously described (33) and verified (Fig. S1B).

The calculation of individual IgG concentrations for each influenza strain anti-HA IgG was performed using standard curves generated from five-parameter logistic regression models (27, 28). All IgG concentration results from the mPlex-Flu assay were adjusted using linear mixed-effects models accounting for the group, day, and group-day interactions for each H5 vaccine strain. Covariates adjusted in the linear mixed-effects models included age at enrollment, gender, ethnicity (Caucasian versus non-Caucasian), dose (two dose levels, 15 and 90 μg), and analytic batch (five batches) factors (27, 28).

### Antigenic cartography of H5 influenza viruses generated by mPlex-Flu assay data.

In order to estimate the antigenic distance of HA antigens of H5 influenza virus strains, we adopted the 17 H5 HA genes that covered all 10 clades/subclades strains of H5 from Paul Zhou from the Institute Pasteur of Shanghai, Chinese Academy of Sciences, Shanghai, China (1). The 17 individual antisera against each H5 influenza virus strain were generated with mouse DNA vaccination as previously described (1) and are shown in Fig. S2A. Using the mPlex-Flu assay, we evaluated the 17 antisera against a panel of 36 HA antigens to create a multidimensional matrix, after normalizing the dilution factors and subtracting the background levels, using generalized linear models with identity link functions (Fig. S2B). Classical multidimensional scaling was used to project multidimensional distances into two-dimensional antigenic cartography plots (25, 29). The coordinates for two-dimensional antigenic cartography were further used to calculate the Euclidean distance between H5 influenza viruses to obtain the antigenic distance matrix (Fig. S3).

### Relative antigenic landscapes of the antibody response.

Based on the antigenic distances generated above and using the three vaccine strains HK97 (clade 0), Vie04 (clade 1), and Ind05 (clade 2) as references, a vaccine strain-relative antigenic-distance matrix was selected. Next, relative antigenic antibody landscape-like figures were created by using the relative antigenic distance as the *x* axis and the IgG antibody response as the *y* axis. Data points were linked by LOWESS fit spline curves (Prism 8 software). A set of antibody response landscape-like plots were generated for each vaccination strain.

### H5 head- and stalk-specific antibody response.

We used the mPlex-Flu assay to simultaneously assess the antibodies to the head and stalk domains of HA. We coupled Luminex beads with the head region of HA, which are purified recombinant proteins of the HA1 domains of Ind05 and H9/A/guinea fowl/Hong Kong/WF10/1999 (gfHK99, H9). To detect the group 1 stalk-reactive antibodies, we used the chimeric cH5/H1 (head/stalk) and cH9/H1 proteins. For group 2 stalk-reactive antibodies, we used the cH5/H3 and cH7/H4 proteins kindly provided by Florian Krammer (31, 33, 34, 52).

### Reanalyses of HAI and MN data.

Primary HAI and MN data were generated previously during the vaccine trial as described previously (24). Serum antibody responses to the homologous A/Indonesia/05/2005 PR8-IBCDC-RG2 virus were measured at the Southern Research Institute (6). We reanalyzed these data using linear mixed-effects models, with correlations from repeated measurements within the same subject considered. The same predictors and covariates were used in the linear mixed-effects models for the HAI and MN data analysis as for the mPlex-Flu data analysis (27).

### Data availability.

All data generated in this study are included in this published article and in the supplemental material.

10.1128/mBio.00449-21.1 TEXT S1 Supplemental materials and methods. Download Text S1, PDF file, 0.1 MB. Copyright © 2021 Wang et al. 2021 Wang et al. https://creativecommons.org/licenses/by/4.0/ This content is distributed under the terms of the Creative Commons Attribution 4.0 International license.
