# Supplementary figures and images for: Broadly Reactive IgG Responses to Heterologous H5 Prime-Boost Influenza Vaccination Are Shaped by Antigenic Relatedness to Priming Strains
Source: mBio. 2021 Jul 6;12(4):e00449-21. doi: 10.1128/mBio.00449-21 (PMC8406322; doi:10.1128/mBio.00449-21)

A

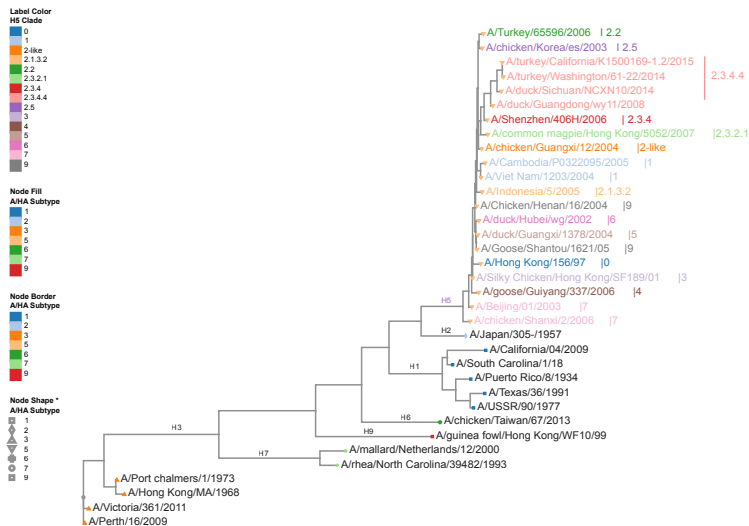

B

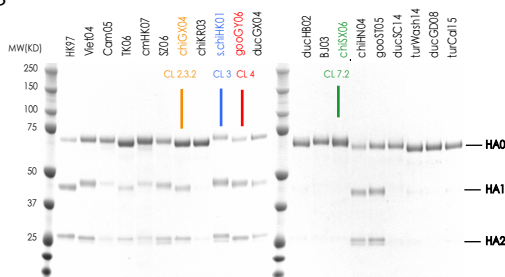

C

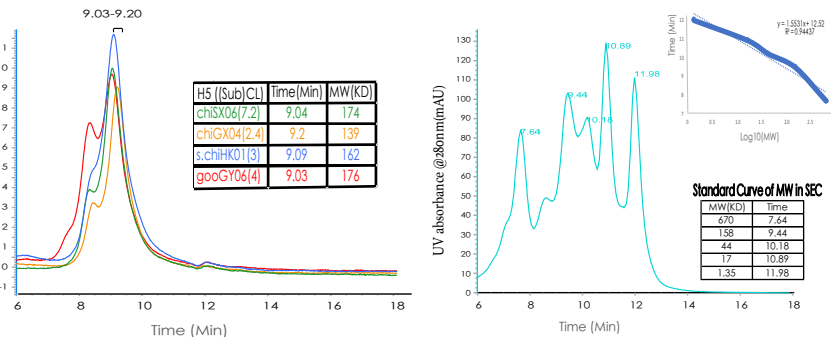

Supplement: FIG S1 [file mbio.00449-21-sf001.pdf]
