# Supplementary material for: Broadly Reactive IgG Responses to Heterologous H5 Prime-Boost Influenza Vaccination Are Shaped by Antigenic Relatedness to Priming Strains
Source: mBio. 2021 Jul 6;12(4):e00449-21. doi: 10.1128/mBio.00449-21 (PMC8406322; doi:10.1128/mBio.00449-21)
Supplement: TABLE S1 [file mbio.00449-21-st001.pdf]

Table S1. The mPlex-Flu assay panel of seasonal influenza viruses, H5 clades and subclades

Influenza Virus Type	Subtypes	Full Name of Viruses	Abbreviation	H5 Clades /Subclades	Genbank Accession #
A	H1	A/South Carolina/1/18	SC18		AF117241.1
		A/Puerto Rico/8/1934	PR8		CY148243.1
		A/USSR/90/1977	USSR77		DQ508897.1
		A/New Caledonia/20/1999	NewCall99		DQ508889.1
		A/Texas/36/1991	Tex91		CY125100.1
		A/California/07/2009	Cali09		FJ966974.1
	H2	A/Japan/305/1957	Jap57		L20407.1
	H3	A/Port Chalmers/1/1973	PC73		CY112249.1
		A/Hong Kong/1/1968	HK68		CY009348.1
		A/Perth/16/2009	Per09		GQ293081.1
		A/Victoria/361/2011	Vic11		KM821347
		A/Texas/50/2012	Tex12		KC892248.1
	H5	A/Hong Kong/156/97	HK97	0	AF028709
		A/Viet Nam/1203/2004	Viet04	1	EF541403
		A/Cambodia/P0322095/2005	Cam05	1.1	HQ200458
		A/Indonesia/5/05	Ind05	2.1.3.2	EF541394
		A/Turkey/65596/2006	TK06	2.2.1	EF619998
		A/Common Magpie/Hong Kong/5052/2007	cmHK07	2.3.2.1	CY036173
		A/Shenzhen/406H/2006	SZ06	2.3.4	EF137706
		A/Chicken/Guangxi/12/2004	chiGX04	2.4	DQ366330
		A/Chicken/Korea/es/2003	chiKR03	2.5	EF541412
		A/Silky Chicken/Hong Kong/SF189/01	s.chiHK01	3	AF509021
		A/Goose/Guiyang/337/2006	gooGY06	4	DQ992765
		A/Duck/Guangxi/1378/2004	ducGX04	5	DQ320884
		A/Duck/Hubei/wg/2002	ducHB02	6	DQ997094
		A/Beijing/01/2003	BJ03	7.1	EF587277
		A/Chicken/Shanxi/2/2006	chiSX06	7.2	DQ914814
		A/Chicken/Henan/16/2004	chiHN04	8	AY950234
		A/Goose/Shantou/1621/05	gooST05	9	DQ095628
		A/duck/Sichuan/NCXN10/2014	ducSC14	2.3.4.4	KM251469
		A/turkey/Washington/61-22/2014	turWash14	2.3.4.4	KP739397
		A/duck/Guangdong/wy11/2008	ducGD08	2.3.4.4	CY091627
		A/turkey/California/K1500169-1.2/2015	turCal15	2.3.4.4	KR150901
	H6	A/Taiwan/2/2013	TW13		KJ162860.1
	H7	A/mallard/Netherlands/12/2000	malNert00		EF470586
		A/rhea/North Carolina/39482/1993	rheaNC93		KF695239
	H9	A/guinea fowl/Hong Kong/WF10/1999	gfHK99		AY206676.1
HA domains		Head of A/Indonesia/5/05	H5 Head		
		Head of A/guinea fowl/Hong Kong/WF10/1999	H9 head		
Chimeric HA		cH5/1 (A/Indonesia/5/05, A/Puerto Rico/8/1934)	cH5/1PR		
		cH5/1 (A/Indonesia/5/05, A/California/07/2009)	cH5/1Cal		
		cH9/1 (A/gf/HK/WF10/1999, A/California/07/2009)	cH9/1		
		cH4/7 (A/duck/Czech/1956, A/Shanghai/1/2013)	cH4/7		
