# Supplementary material for: Broadly Reactive IgG Responses to Heterologous H5 Prime-Boost Influenza Vaccination Are Shaped by Antigenic Relatedness to Priming Strains
Source: mBio. 2021 Jul 6;12(4):e00449-21. doi: 10.1128/mBio.00449-21 (PMC8406322; doi:10.1128/mBio.00449-21)
Supplement: TEXT S1 [file mbio.00449-21-s0001.pdf]

Supplementary Material

Generation of an antigenic cartography representing 21 clades or subclades of H5 influenza viruses using mouse antisera reactivity measured using our mPlex-Flu assay

1. ANIMALS

Female BALB/c mice were purchased from Taconic Biosciences. For all experiments, female 8- to 12-week-old mice were used and randomly assigned to experimental groups. All research involving live, vertebrate animals was conducted in accordance with the Public Health Service Policy on Human Care and Use of Laboratory Animals. mice were maintained at the University of Rochester Medical Center Vivarium, a AAALAC certified Vivarium (Animal Welfare Assurance Number is A-3292-01), under their established guidelines, including isolation, feeding, recovery procedures, and euthanasia in accordance with Federal regulations. All experimental procedures for animals were approved by the Institutional Animal Care and Use Committee (IUCAC; protocol number UCAR-2011-055E), and all personnel working with the animals were trained and certified by the IUCAC and Vivarium staff.

2. GENERATION OF MOUSE ANTISERA DIRECTED AGAINST HEMAGGLUTININS (HAS) REPRESENTING H5 CLADES AND SUBCLADES

A panel of 17 total DNA plasmids encoding all 10 H5 clades/subclades HA genes was provided by Dr. Paul Zhou from Institute Pasteur of Shanghai, Chinese Academy of Sciences, Shanghai, China (Zhou et al., 2012). The DNA vaccination plamids were constructed from the mammalian expression vector, pCMV/R, containing whole codon-optimized HA gene inserts representing H5 influenza viruses (Zhou et al., 2012). All above plamid DNAs were amplified and then purified with Plasmid Maxi Kit (QIAGEN) following the manufacture's recommendations. Purified DNA plamids were used for intramuscular immunizations (i.m.) of 8- to 12-week-old BALB/c mice as previously described (Zhou et al., 2012). Briefly, the mice (n=4) were inoculated with 100g of one of the 17 H5 subclade HA plamid DNAs respectively, on days 0, 28 and 56 (see Figure S2 A). Fourteen days after the last immunization, serum samples were collected from each mouse and combined within each group. The resultant H5 reactive antisera panel was aliquoted and stored at -20°C for future analysis.

3. RECOMBINANT HA PROTEINS (RHAS) OF H5 CLADES AND SUBCLADES

All rHAs of type A and B influenza viruses used in this study (TABLE S1) were expressed using pFastBac baculovirus system with a C-terminal trimerization domain and a hexahistidine purification tag (Wang et al., 2018). The entire panel of 17 H5 clades and subclades HA genes were subcloned into this pFastBac vector using BamHI and NotI restriction endonucleases (NEB, Ipswich, MA) as previously published (Wang et al., 2018). We also synthesized four HA genes (see TABLE S1) of H5 avian influenza viruses belonging to the new subclade 2.3.4.4 that circulated during the huge H5 outbreak in turkey and chicken farms in USA in 2015, including A/duck/Sichuan/NCXN10/2014 (ducSC14,gene bank accession No:KM251469), A/turkey/Washington/61-22/2014 (turWash14,accession No:KP739397), A/duck/Guangdong/wy11/2008 (ducGD08, accession No:CY091627) and A/turkey/California/K1500169-1.2/2015 (turCal15, accession No: KR150901). We also inserted these HA genes into the pFastBac expression vector to express the rHAs of H5 influenza virus HAs.

Expression and purification of rHA was performed as previously described (Wang et al., 2018). Purified rHAs were concentrated and desalted with 30 kDa Amicon Ultracell centrifugation units (Millipore, Billerica, MA) and re-suspended in phosphate buffered saline (PBS, pH7.4). The purity, integrity and identity of proteins was assessed by NuPage 4-12% Bis-Tris gels (Invitrogen, Grand Island, NY), the results of which are shown in FIG S1 B. Protein concentration was quantified using the Quickstart Bradford Dye Reagent (Bio-Rad, Hercules, CA) with a bovine serum albumin standard curve.

4. SIZE EXCLUSION CHROMATOGRAPHY (SEC) ANALYSIS OF RHAS OF H5 INFLUENZA VIRUSES

The four representative rHAs of H5 influenza viruses were evaluated, including A/Chicken/Guangxi/12/2004 (chiGX04, CL2.4), A/Silky Chicken/Hong Kong/SF189/01 (s.chiHK01, CL3), A/Goose/Guiyang/337/2006 (gooGY06, CL4) and A/Chicken/Shanxi/2/2006 (chiSX06, CL7.2) (Colored in FIG S3. A). Partially cleaved HA0 (into HA1 and HA2) and uncleaved HA0 were analyzed by SEC using the AKTA chromatography system (GE Healthcare Bio-Sciences, Pittsburgh, PA) through a HPLC Biosep-SEC-s4000 column (300X7.8mm, 00H-2147-K0, Phenomenex Inc, Torrance, CA) in pH 6.8 buffer of 50mM Na2HPO4, 50mM NaHPO4 and 150mM NaCl at 1.0 ml/min flow rate. All four purified HA preps took the same length of time to flow out from SEC column in HPLC analysis (see FIG S1 C), suggesting that there are not significant differences between the sizes of the protein preps, regardless of whether the HA is uncleaved, partially or fully cleaved. This result provides evidence that the cleavage of HA0 does not affect HA1 and HA2 binding to form the trimmer structure in the natural condition. The protein standards for SEC were purchased from Bio-Rad (151-1901, Bio-Rad Inc, Hercules, CA), and the peaks of protein standards in HPLC analysis are shown in FIG S1 C.

5. DEVELOPMENT OF H5 MPLEX-FLU ASSAY

The mPlex-Flu assay contained an HA antigen panel with 35 rHAs from various influenza strains, HA domains and chimeric HAs (Supplementary Table S1). The phylogenetic amino acid sequence tree of those 35 HA proteins is shown in FigureS1.

We used the mPlex-Flu assay to estimate the strain specific binding of the 17 H5 DNA vaccination anti-sera with our 35 HA antigen panel as previously described (Wang et al., 2018). After normalizing the dilution factor using the generalized linear model with identity link function, strain specific binding was obtained from the estimated coefficients, using the background binding as the reference group (i.e. the strain specific binding is equal to the estimated strain binding minus the background binding).

6. GENERATION AN ANTIGENIC CARTOGRAM UTILIZING A NOVEL MULTIPLE DIMENSIONAL SCALING METHOD (MDS)

Multidimensional scaling preserves the dissimilarities among strains by generating a two-dimension plot with the distance between each strain approximating their dissimilarities. Using classical (metric) MDS, multiple dimensional matrix data were generated using mPlex-Flu assay data, as shown in Figure S2, B. The resultant two-dimensional MDS plot allowed us to estimate the H5 HA antigenic distances and generate the HA antigenic cartography as previously published (Zhou et al., 2012; Smith et al., 2004). Due to the continuous nature of data from the mPlex-Flu assay (as compared to the relative discrete data from the HAI assay) and the consistent range of estimated strain specific binding, we constructed the antigenic map by minimizing different error functions $E = \sum_{ij} e(MFI_{ij}, d_{ij})$, where MFI_{ij} denotes the target distance between antigen i and antiserum j and d_{ij} denotes the Euclidean distance between antigen i and antiserum j in the two-dimensional map. $e(MFI_{ij}, d_{ij}) = (MFI_{ij} - d_{ij})^2$ is the error function we minimized to construct the antigenic cartogram.

We also conducted sensitivity analysis to compare the generated antigenic cartography with previous published methods (Smith et al., 2004). We replaced $MFI^*_{ij} = b_j - MFI_{ij}$ with MFI_{ij} as input for the MDS method, where b_j denotes the maximum measurement for antiserum j. We obtained the same antigenic cartography as expected due to the consistent range of the estimated strain specific binding, shown in Figure S2, C.

An HA antigenic cartography consisting of 21 H5 influenza virus strains was generated utilizing antisera from H5 DNA vaccinated mice, to calculate the antigenic distance from the H5 A/Hong Kong/1997 clade 0 (HK97(0)), see Supplementary Figure S3.

7. FIGURES AND TABLES

Supplementary Figure 1: HA protein characters of 35 influenza virus A strains in mPlex-Flu assay. A. The phylogenetic tree was generated using HA amino acid sequences of the 35 influenza A virus strains obtained from the phylogenic tree maker on the Influenza Research Database Website (https://www.fludb.org/brc/home.spg?decorator=influenza). B.SDS-PAGE gel image of purified HA proteins of H5 influenza viral strains. C. HPLC analysis results of four representative HA proteins flowing through the Biosep-SEC-s4000 columns with the Bio-rad protein standards.

Supplementary Figure 2: Antigenic cartography is generated with a mouse DNA vaccination model. A. Mouse DNA vaccination strategy. B. Heat map of the multiple dimensional antibody data generated by the mPlex-Flu assay. Each mouse polyclonal antiserum was induced by DNA vaccination with a DNA plasmid encoding HA proteins, and the antibody levels in the sera were estimated by mPlex-Flu assay. C. Antigenic cartography of 36 influenza A strains assessed by mPlex-Flu assay with the Multiple Dimensional Scaling (MDS) method.

Supplementary Figure 3: The heat-map matrix of the antigenic distance between the 21 H5 influenza virus strains. The three vaccination strains are highlighted with red arrows.

Supplementary Figure 4: The correlation between the HA antibody response and HA antigenic similarity of A/Hong Kong/156/97 (HK97) to 21 H5 influenza virus strains. A. The HA antibody response landscape-like plots of each group using the relative HA antigenic distance of A/Hong Kong/156/97 (HK97, clade 0) as the reference strains (see material and methods). X-axis is relative antigenic distance; Y-axis is IgG antibody response; the spots were linked by LOWESS fit spline curve (Prism 8 software). B. The correlation of the HA antibody response to the HA-antigenic distance. The Δ change of antibody concentration of pre- and post- vaccination versus the relative HA antigenic distance of Vie04. The R squared values were calculated with simple linear regression analysis (Prism 8 software).

Supplementary Figure 5:The correlation between the HA antibody response and HA antigenic similarity between A/Vietnam/1203/2004 (Vie04) and 21 H5 influenza virus strains. A. The HA antibody response landscape-like plots of each group using the relative HA antigenic distance of A/Vietnam/1203/2004 (Vie04, clade 1) as the reference strains (see material and methods). X-axis is relative antigenic distance; Y-axis is IgG antibody response; the spots were linked by LOWESS fit spline curve (Prism 8 software). B. The correlation of the HA antibody response to the HA-antigenic distance. The Δ change of antibody concentration of pre- and post- vaccination versus the relative HA antigenic distance of Vie04. The R squared values were calculated with simple linear regression analysis (Prism 8 software).

Supplementary Figure 6: The correlation between the HA antibody response and HA antigenic similarity of A/Indonesia/5/05 (Ind05) to 21 H5 influenza virus strains. A. The HA antibody response landscape-like plots of each group using the relative HA antigenic distance of A/Indonesia/5/05 (Ind05, clade 1) as the reference strains (see material and methods). X-axis is relative antigenic distance; Y-axis is IgG antibody response; the spots were linked by LOWESS fit spline curve (Prism 8 software). B. The correlation of the HA antibody response to the HA-antigenic distance. The Δ change of antibody concentration of pre- and post- vaccination verse the relative HA antigenic distance of Vie04. The R squared values were calculated with simple linear regression analysis (Prism 8 software).

Supplementary Figure 7: The IgG concentration of group 1 and 2 influenza virus strains was estimated by mPlex-Flu assay in the DMID 08-0059 study. The mPlex-Flu assay estimated the mean and standard deviation of IgG concentration for each group. Then the antibody concentrations were adjusted within the linear mixed-effects models, which included the following: age at enrollment, gender, ethnicity (Caucasian vs. non-Caucasian), dose (two dose levels: 15 and 90 μg), and batch (five batches). A. The mPlex-Flu assay estimated the antibody concentrations of group 1 influenza virus strains (including five human H1, one of each H2, H6, and H9). B. The antibody concentrations to group 2 influenza A virus strains (including four H3, and two H7 strains) were estimated by the mPlex-Flu assay.

Supplementary Figure 8: Prior vaccination with a monovalent influenza vaccine (MIV) increased the serum titers of hemagglutination-inhibition (HAI) and micro-neutralization (MN) antibody responses against three antigenically drifted virus vaccine strains, including new vaccine strain A/Indonesia/05/2005 (Ind05; clade 2), previous MIV strains A/Vietnam/1203/2004 (Vie04; clade 1), A/Hong Kong/156/1997 (HK97; clade 0). Naive subjects (Unprimed) received the MIV Ind05 strain and were subsequently boosted at day 28 with the same strain. A previous primed group, received the MIV Vie04 5 years prior, (Primed) then received a single dose of Ind05. The previous double primed MIV Vie04 and HK97 (Multiple). The mean and standard deviation

of IgG concentration for each group were estimated by linear mixed effects models with group, day, and group-day interaction used to fit the data for each H5 vaccine strain. Covariates adjusted in the linear mixed effects models included the following: age at enrollment, gender, ethnicity (Caucasian vs. non-Caucasian), dose (two dose levels: 15 and 90 μg), and batch (five batches). * P<0.05, **P<0.01, ***P<0.001 Linear contrasts within the linear mixed effects models framework were used to do the statistical testing.

Supplementary Table 1: The mPlex-Flu assay panel of seasonal influenza viruses, H5 clades and subclades.

REFERENCES

Smith, D. J., Lapedes, A. S., de Jong, J. C., Bestebroer, T. M., Rimmelzwaan, G. F., Osterhaus, A. D., & Fouchier, R. A. (2004). Mapping the antigenic and genetic evolution of influenza virus. *Science*, *305*(5682), 371–6.
URL https://www.ncbi.nlm.nih.gov/pubmed/15218094

Wang, J., Hilchey, S. P., DeDiego, M., Perry, S., Hyrien, O., Nogales, A., Garigen, J., Amanat, F., Huertas, N., Krammer, F., Martinez-Sobrido, L., Topham, D. J., Treanor, J. J., Sangster, M. Y., & Zand, M. S. (2018). Broad cross-reactive igg responses elicited by adjuvanted vaccination with recombinant influenza hemagglutinin (rha) in ferrets and mice. *PLoS One*, *13*(4), e0193680.
URL https://www.ncbi.nlm.nih.gov/pubmed/29641537

Zhou, F., Wang, G., Buchy, P., Cai, Z., Chen, H., Chen, Z., Cheng, G., Wan, X. F., Deubel, V., & Zhou, P. (2012). A triclade dna vaccine designed on the basis of a comprehensive serologic study elicits neutralizing antibody responses against all clades and subclades of highly pathogenic avian influenza h5n1 viruses. *J Virol*, *86*(12), 6970–8.
URL https://www.ncbi.nlm.nih.gov/pubmed/22496212
